# *Wolbachia* and DNA Barcoding Insects: Patterns, Potential, and Problems

**DOI:** 10.1371/journal.pone.0036514

**Published:** 2012-05-02

**Authors:** M. Alex Smith, Claudia Bertrand, Kate Crosby, Eldon S. Eveleigh, Jose Fernandez-Triana, Brian L. Fisher, Jason Gibbs, Mehrdad Hajibabaei, Winnie Hallwachs, Katharine Hind, Jan Hrcek, Da-Wei Huang, Milan Janda, Daniel H. Janzen, Yanwei Li, Scott E. Miller, Laurence Packer, Donald Quicke, Sujeevan Ratnasingham, Josephine Rodriguez, Rodolphe Rougerie, Mark R. Shaw, Cory Sheffield, Julie K. Stahlhut, Dirk Steinke, James Whitfield, Monty Wood, Xin Zhou

**Affiliations:** 1 Department of Integrative Biology and the Biodiversity, Institute of Ontario, University of Guelph, Guelph, Ontario, Canada; 2 Department of Biology, Dalhousie University, Halifax, Nova Scotia; 3 Natural Resources Canada, Canadian Forest Service, Atlantic Forestry Centre, Fredericton, New Brunswick, Canada; 4 Department of Entomology, California Academy of Sciences, San Francisco, California, United States of America; 5 Department of Entomology, Cornell University, Ithaca, New York, United States of America; 6 Department of Biology, University of Pennsylvania, Philadelphia, Pennsylvania, United States of America; 7 University of New Brunswick, Fredericton, New Brunswick Canada; 8 Biology Center, Czech Academy of Sciences and Faculty of Science, University of South Bohemia, Ceske Budejovice, Czech Republic; 9 Institute of Zoology, Chinese Academy of Sciences, Beijing, China; 10 Museum of Comparative Zoology, Harvard University, Cambridge, Massachusetts, United States of America; 11 Department of Biology, York University, Toronto, Ontario, Canada; 12 Division of Biology, Imperial College London, Silwood Park Campus, Ascot, Berkshire, United Kingdom; 13 Department of Entomology, Natural History Museum, London, United Kingdom; 14 National Center for Ecological Analysis and Synthesis (NCEAS) University of California Santa Barbara, Santa Barbara, California, United States of America; 15 Université de Rouen, laboratoire ECODIV, Mont Saint Aignan, France; 16 National Museums of Scotland, Edinburgh, United Kingdom; 17 University of Illinois, Department of Entomology, Urbana, Illinois, United States of America; 18 Diptera Unit, Canadian National Collection of Insects, Arachnids and Nematodes, Agriculture and Agri-Food Canada, Ottawa, Ontario, Canada; 19 Beijing Genomics Institute (BGI) BGI-Shenzhen, Shenzhen, Guangdong Province, China; J. Craig Venter Institute, United States of America

## Abstract

*Wolbachia* is a genus of bacterial endosymbionts that impacts the breeding systems of their hosts. *Wolbachia* can confuse the patterns of mitochondrial variation, including DNA barcodes, because it influences the pathways through which mitochondria are inherited. We examined the extent to which these endosymbionts are detected in routine DNA barcoding, assessed their impact upon the insect sequence divergence and identification accuracy, and considered the variation present in *Wolbachia* COI. Using both standard PCR assays (*Wolbachia* surface coding protein – *wsp*), and bacterial COI fragments we found evidence of *Wolbachia* in insect total genomic extracts created for DNA barcoding library construction. When >2 million insect COI trace files were examined on the Barcode of Life Datasystem (BOLD) *Wolbachia* COI was present in 0.16% of the cases. It is possible to generate *Wolbachia* COI using standard insect primers; however, that amplicon was never confused with the COI of the host. *Wolbachia* alleles recovered were predominantly Supergroup A and were broadly distributed geographically and phylogenetically. We conclude that the presence of the *Wolbachia* DNA in total genomic extracts made from insects is unlikely to compromise the accuracy of the DNA barcode library; in fact, the ability to query this DNA library (the database and the extracts) for endosymbionts is one of the ancillary benefits of such a large scale endeavor – for which we provide several examples. It is our conclusion that regular assays for *Wolbachia* presence and type can, and should, be adopted by large scale insect barcoding initiatives. While COI is one of the five multi-locus sequence typing (MLST) genes used for categorizing *Wolbachia*, there is limited overlap with the eukaryotic DNA barcode region.

## Introduction

DNA barcoding uses a standardized short sequence of DNA as a key character for species-level identification and discovery [1]. Barcode variation can be used for the identification of known species from trace amounts of tissue [2] or a taxonomically unidentifiable stage [3] or as a part of a suite of characters for the discovery and description of new species [4]. As a tool in revisionary studies it can speed up the rate of taxonomic research in flagging otherwise cryptic diversity [1], [5]–[7]. Within arthropods, the approach has been used in many orders [1], [4], [5], [7]–[11] utilizing the mitochondrial cytochrome *c* oxidase subunit 1 (COI) gene with reports of success and of failure [12], [13]. In some cases where it has failed – when there was not sufficient variation present in the barcode region to differentiate between species [11] or where there was an evident mito-nuclear discordance such that intra-specific mtDNA variation might be confused with inter-specific variation [14], [15] – the failures were hypothesized to be due to the effects of the host-manipulating intracellular rickettsial-type symbiotic bacteria, *Wolbachia*.

*Wolbachia* are alpha-proteobacterial reproductive parasites which can alter the sex-ratio and reproductive compatibility of their host to their own benefit [16]. They are among the most common endosymbiotic bacteria in many, perhaps most, arthropod systems. Known effects of *Wolbachia* include cytoplasmic incompatibility (CI) in which matings between uninfected females and infected males produce inviable embryos, and male-killing (MK) in which infected females produce no (or a reduced number of) viable male offspring. These strategies generally increase the reproductive success of infected relative to uninfected matrilines. Perhaps the best known, and/or most frequently reported impact of *Wolbachia* on its host behavior is CI. In CI, any zygote formed through fertilization of an uninfected egg with sperm from an infected male dies. This strategy of host manipulation has been remarkably successful and it has been estimated that as many as 66% of all insect species carry a *Wolbachia* infection [17], although *Wolbachia* incidence is not the same as CI prevalence. This favoring of infected matrilines can also drive a mitochondrial sweep through a population (or species), confounding interpretations of mtDNA divergence among populations as outlined below [18].

Infections of bacterial endosymbionts could threaten the accuracy of an mtDNA based system of identification and species discovery such as DNA barcoding in any one of four ways:

1. Unintended amplification of bacterial COI due to the use of broad, near-universal primer sets and failure to then recognize these sequences as bacterial.
2. Conflation or confusion of insect species identifications due to the inclusion of the bacterial endosymbiont COI.
3. Lineage disruption via CI as an isolating mechanism leading to the conflation of insect lineages that are infected with different *Wolbachia* strains within a species (thereby overestimating diversity; i.e. individuals within a population being swept with a mitochondrial type via a *Wolbachia* infection may appear as different species using mtDNA barcoding).
4. Lineage disruption via CI as an isolating mechanism leading to the fixation of one species' mtDNA within a hybridizing species pair for which one carries a *Wolbachia* infection (underestimating diversity; i.e. hybridization resulting in the replacement of the mitochondria of one species with that of the other [19], [20]).

*Wolbachia* can be amplified from arthropod total genomic DNA extracts made from somatic tissue [21] (including legs, the most commonly used material for DNA barcoding projects). We have demonstrated this previously utilizing the *Wolbachia* surface protein coding gene (*wsp*) assay [22] to test for endosymbiont prevalence within certain groups being assayed for DNA barcode variation (Formicidae – [4], [7]; Tachinidae – [11], [23]; Braconidae – [10]). We have also experienced the un-intended amplification of *Wolbachia* COI from insect genomic DNA extracts [7]. We examined the more than two million insect trace files on the Barcode of Life Datasystem (BOLD - [24]) for evidence of un-intended amplification of *Wolbachia* and also conducted more in-depth cases studies using more than 95K DNA extracts from three insect orders (Hymenoptera, Diptera, and Lepidoptera) and more than nine families to ask 1) whether these unintended amplifications would compromise our capacity to generate or analyze the barcodes of their insect hosts; 2) whether the observed frequency of *Wolbachia* COI amplification is a function of *Wolbachia* prevalence as measured using the *wsp* PCR assay; and 3) what *Wolbachia* phylogenetic information can be gleaned from bacterial gene regions generated from insect DNA barcoding surveys.

We conclude that unrecognised amplification of bacterial COI or the confusion of insect identifications due to the inclusion of unanticipated amplification of bacterial COI does not represent a serious impediment for a barcoding survey of a taxon or area. Such incidences are rare and can be easily recognized if queried. Our greatest concern *a priori* regarding the potential effects of *Wolbachia* on mtDNA based identifications, and on species discovery, was the potential conflation of infected (and isolated) lineages within species as species – but we have not yet documented such a case. A DNA barcoding survey through a taxon or sampling regime is far from being compromised by the influence of *Wolbachia*. Rather, these surveys represent an ideal opportunity to explore what relationships actually do exist between different bacterial strains and hosts and between bacteria from different hosts in different geographic regions.

## Results

### Unanticipated amplification of bacterial COI from insect hosts and primer specificity

The Barcode of Life Data System (BOLD- [24]) library of trace files was searched for evidence of *Wolbachia* (*Wolbachia* is one of the suite of possible contaminants that all sequences uploaded to BOLD are checked against as a normal quality-control routine - [24]). Out of 1.09 million insect specimen trace files searched, generated from extractions principally (but not exclusively) based on somatic tissue, we found evidence of *Wolbachia* in 1,768 traces (0.16%). Non-specific amplification of *Wolbachia* was found in multiple insect orders (Table 1) and using multiple primer combinations (Table 2), however, that amplicon was never confused with the COI of the host.

**Table 1:** Ordinal table where trace search of BOLD contained specimens where at least one trace file contained an un-intended *Wolbachia* amplification.

Order	Specimens where at least one amplification produced a *Wolbachia* amplicon	Specimens with sequences (BOLD taxonomy Browser)	Proportion
Hymenoptera	1378	140,613	0.98%
Lepidoptera	268	539,174	0.05%
Diptera	55	102,139	0.05%
Hemiptera	18	21,283	0.08%
Araneae	17	24,361	0.07%
Coleoptera	12	31,281	0.04%
Poduromorpha	3	4,227	0.07%
Trombidiformes	3	3,546	0.08%
Dermaptera	2	131	1.53%
Odonata	2	5,044	0.04%
Orthoptera	2	5,276	0.04%
Trichoptera	2	30,184	0.01%
Ephemeroptera	1	8,946	0.01%
Psocoptera	1	332	0.30%
Sarcoptiformes	1	3,390	0.03%
Symphypleona	1	986	0.10%

**Table 2:** Primer pairs involved in the unanticipated recovery of bacterial COI from insect DNA extracts. All Primer codes, and oligo sequences, are available on BOLD (www.barcodinglife.org).

Primer Pair (Forward/Reverse)	Percentage of *Wolbachia* present in BOLD trace search
C_LepFolF/C_LepFolR	1.36%
C_tRWFt1/LepR1	0.06%
C_VF1LFt1/C_VR1LRt1	0.06%
HCO2198_t1/LCO1490_t1	0.03%
LCO1490/HCO2198	0.34%
LCO1490_t1/HCO2198_t1	2.45%
LepF1/C_ANTMR1D	0.03%
LepF1/EnhLepR1	0.81%
LepF1/LepR1	86.77%
LepF1/MLepR1	1.70%
LepF2_t1/LepR1	0.22%
LepR1/LepF1	0.03%
MLepF1/LepR1	0.03%
MLepR1/LepF1	0.03%
OdoF1_t1/OdoR1_t1	0.06%
RonM_t1/LepR1	0.03%
RonMWASPdeg_t1/LepR1	5.89%
T-LepF1-short/T-LepR1-short	0.06%

For example, within Lepidoptera there are, at the time of writing, more than 506,297 COI DNA barcodes on BOLD (BOLD Taxonomy Browser, Lepidoptera sequences on BOLD on June 2011) within which we found only 286 cases where *Wolbachia* COI was amplified rather than the insect (0.05%) (as of June 2011). For those Lepidoptera generated as part of the Área de Conservación Guanacaste (ACG) rearing and light-collecting program [1] we found 186 *Wolbachia* sequences from the 162,065 specimens of ACG Lepidoptera barcoded (BOLD Taxonomy Browser on 11.06.02) – 0.11%)).

### Conflation of insect identifications due to the inclusion of the bacterial endosymbiont COI

On average, there are 167 base pair differences between *Wolbachia* and their host COI within the barcode region. Bacterial COI GC content does not possess the characteristic insect AT bias (Table 3 - the average GC content of the insect hosts is 13%, while in *Wolbachia* it is much higher (20%)).

**Table 3:** Comparison between insect host and endosymbiotic bacteria COI for 255 specimens. (nucleotide content and variability within and between each group).

	*Wolbachia*	HOST		Pairwise distances	Pairwise distances
	Mean	Mean	Group	within group	between group
G %	20.31	13	*Wolbachia*	5.65	
C %	18.06	12.86	HOST	84.31	167.96
A %	23.72	30.35			
T %	37.44	42.67			

### *wsp* assay and prevalence

For three sub-sets of data (ants from the south-western Indian Ocean island of Mauritius, and both ants and parasitoid wasps from Churchill, Manitoba, Canada – Table 4) we used the PCR based wsp assay [22] to test whether the proportion of generated bacterial COI was correlated with the frequency of *Wolbachia* in the insects themselves. A subset of these bands was sequenced to confirm the identity of the surface coding protein.

**Table 4:** Case studies of the frequency of bacterial COI and *wsp* recovered from whole genomic DNA extracts made from 10 insect families from two orders. [46],[47].

Case Study	Families	Target CO1 sequences (Insect)	Non-target CO1 sequences (*Wolbachia*)	Bacterial CO1 as Proportion of Total	wsp PCR Assay (+/total) %	Insect Diversity Examined (# of barcode 2% units)	Total Diversity Infected by CO1 or by WSP (# in italics)	References
ACG Parasitoids	Ichneumonidae Braconidae Tachinidae Chalcididae Eulophidae Pteromalidae	37,333	121	0.32%	Not tested	3,320	18	[10], [11], [23]
Global Ants	Formicidae	31,208	116	0.37%	Not tested	5,316	23	
Ants of Mauritius	Formicidae	1,111	4	0.36%	(116/438) 26.48%	53	*17*	[7]
Malagasy Ants	Formicidae,	175	0	0%	(94/188) 50%	26	*20*	
Parasitoids from French Guiana (abdomens)	Ichneumonidae Braconidae Tachinidae Chalcidae Eulophidae Pteromalidae Trichogrammatidae	553	5	0.90%	Not tested	311	5	
Parasitoids from Belize (abdomens)	Ichneumonidae Braconidae Tachinidae Chalcidae Eulophidae Pteromalidae Trichogrammatidae	919	1	0.11%	Not tested	498	1	
Parasitoids from Ontario Malaise traps (abdomens)	Ichneumonidae Braconidae Tachinidae Chalcidae Eulophidae Pteromalidae Trichogrammatidae	1,793	14	0.78%	Not tested	774	12	
Halicitidae Bees	Halictidae	11,767	443	3.76%	Not tested	808	81	
Parasitoid Wasps of Churchill	Ichneumonidae Braconidae Diapridae Pteromalidae Tenthredinidae	6,749	4	0.06%	(203/376) 53.99%	1,052	86	[5], [25]
Ants of Churchill	Formicidae	442	0	0%	(178/282) 63.12%	6	6	[5]
Parasitoids of New Brunswick	Ichneumonidae Braconidae Tachinidae Chalcidae Eulophidae Pteromalidae Trichogrammatidae	1,524	11	0.07%	Not tested	111	1	[45]
Chinese Fig Wasps	Agaonidae, Pteromalidae, Eurytomidae	2,256	213	9.44%	Not tested	198	38	[46]
TOTAL		95,830	922	0.61%	48.40%	12,473	308	

In ants collected on the island of Mauritius [7], we tested 438 ant specimens from 57 species for *Wolbachia* using the wsp assay and found that approximately a third of these specimens and species tested positive (116/438 specimens = 26.5%, 18/57 species = 31.5%). Of the total ant specimens sequenced from the Mauritius project (1111), only 4 bacterial COI sequences were recovered (0.36% - Table 4). In a smaller set of ants collected in Churchill, Manitoba, Canada [5] we found that 178 of 282 DNA extracts from 5 of 7 species were infected (63%, 71%); however we recovered no bacterial COI from this group using standard insect barcoding procedures.

Using a slightly larger set of parasitoid wasps from Churchill [5], [25], we screened 376 specimens for wsp and found 203 infections (a conservatively estimated rate of infection of almost 54%). However, after sequencing >6,000 parasitoid wasp specimens from Churchill, *Wolbachia* COI was generated only four times in total (0.067%), and never from the 376 specimens that we scanned using wsp primers.

### Comparison to MLST Database

**Figure 1:**
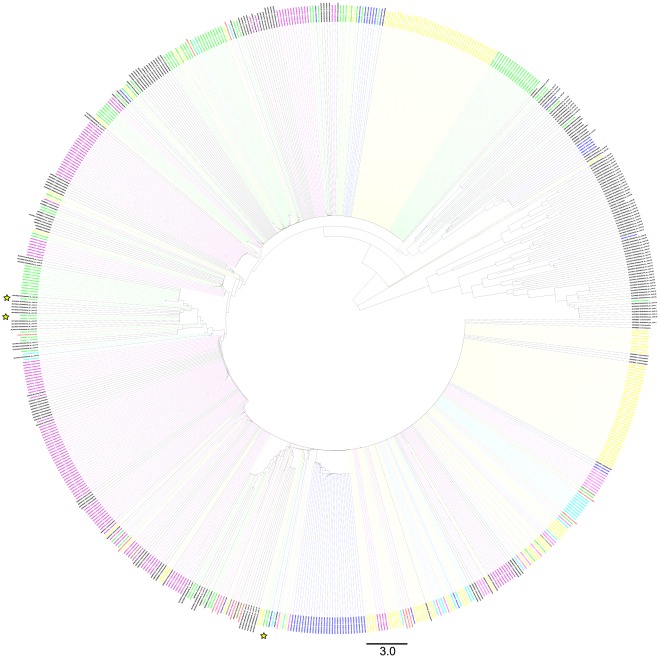
NJ trees based on the 194 bp section of overlap between MLST *Wolbachia* sequences (104) and sequences generated here that have more than 100 bp overlap. Tips labeled by BOLD process ID and host insect taxonomy (if generated here) or MLST allele group. Branches colored by host insect taxonomy (brown = Tachinidae, dark blue = Braconidae, light blue = Halictidae, pink = Chalcididae, red = Ichneumonidae, green = Formicidae, yellow = Lepidoptera, purple = Agaonidae, black = MLST *Wolbachia* alleles). Stars indicate the position of *Wolbachia* from new-world ants.

The multilocus sequence typing (MLST) database [25], [26] allelic profile for COI (or coxA) contains 104 sequences (also see the BOLD project, “MLST – *Wolbachia* from MLST database”). All of the COI *Wolbachia* sequences that have been inadvertently amplified in the insect species we have barcoded are consistent with infections from Supergroup A strains. This indicates a strong bias in amplification towards this supergroup by the insect CO1 primers. Within these Supergroup A strains there are four major allele groups present. One is identical in the overlapping region to the MLST allele coxA-1, a second, to the overlapping region of the allele coxA-6, and two others represent apparently new allele groups (MAS-2, MAS-1) (Table 5, Figure 1 a,b). In only one genus (Hesperiidae, *Urbanus* belliDHJ01, U. belliDHJ03) did we amplify gene fragments consistent with *Wolbachia* Supergroup B (in this case not from COI, but initially using the wsp protocol).

**Table 5:** Comparison of *Wolbachia* allele groups recovered here to the MLST *Wolbachia* allele database with the Family of the host and the range of nations from which the hosts were collected.

Allele group	Frequency	Similarity to MLST	Host Range (Family)	Geographic Range (Nation)	Notes
MAS-2	107	5–11 mismatches to coxA-6	Agaoninae, Agaonidae, Hymenoptera, Sycoryctinae, Sycophaginae, Formicidae, Braconidae, Ichneumonidae, Halictidae,	China, Papua New Guinea, Malaysia, Costa Rica, Canada	52% from Agaoninae, 83% from China
coxA-1	102	99.49% similarty to coxA-1 (1 mismatch)	Agaonidae, Halictidae, Agaoninae, Ichneumonidae, Formicidae, Hymenoptera, Braconidae, Sycophaginae, Chalcididae, Tachinidae, Sycoryctinae	China, Colombia, Costa Rica, Mauritius, United States, Reunion, Canada	22% from Agaonidae, 43% from China. May be as many as three strains -but the variability is outside of MLST region.
coxA-6	97	98.47% similarity to coxA-6 (3 mismatches)	Agaoninae, Epichrysomallinae, Formicidae, Braconidae, Ichneumonidae, Agaonidae, Sycophaginae, Eurytominae, Halictidae, Hymenoptera,	China, Papua New Guinea, United Kingdom, Canada, Costa Rica, Kenya, Madagascar, Thailand	16% from Agaonidae, 72% from China
MAS-1	41	Potential mixture of allelles.	Braconidae, Halictidae, Agaoninae, Formicidae, Hymenoptera, Epichrysomallinae	Costa Rica, United States, China, Papua New Guinea	80% from Braconidae, 68% from Costa Rica
MAS-3 & coxA-17	7	99.49% similarity to coxA-17 (1 mismatch)	Agaoninae, Colletidae, Formicidae	China, South Africa, Zambia, Papua New Guinea, Thailand	
coxA-7 & coxA-19	6	mixture	Ichneumonidae, Chalcididae, Braconidae	Costa Rica, Canada, Papua New Guinea	
coxA-2	5	mixture	Formicidae, Halictidae, Braconidae	Costa Rica, Mauritius, Papua New Guinea	
coxA-111	3	exact	Tachinidae, Agaoninae	Papua New Guinea, China	
coxA-23	2	near hit	Halictidae	Isreal, Kyrgyzstan	
coxA-103 (near)	1	near	Braconidae	Papua New Guinea	
coxA-15 (near)	1	near	Ichneumonidae	Costa Rica	
coxA-33	1	or several others	Ichneumonidae	Costa Rica	
coxA-5	1	or several others	Braconidae	Thailand	
MAS-4	1	99.35% similarity to coxA-44 (1 mismatch)	Formicidae	Papua New Guinea	

## Discussion

One of the first criteria involved in determining a standardized gene region appropriate for a DNA barcoding approach is to find conserved primer regions that enable the utilization of universal (or near-universal) primers [27], [28]. This strategy of near-universal primer design could be compromised if the priming region variability for a taxon in question had less affinity for the barcode oligonucleotide than for a bacterial endosymbiont. In an apparent recent example of this, Linares *et al.* [13] wrote that “… generalized primers led to the inadvertent amplification of the endosymbiont *Wolbachia*, undermining the use of universal primers and necessitating the design of genus-specific COI primers alongside a *Wolbachia*-specific PCR assay.” – and further that, “[t]his result underscores a major problem with the widespread application of universal primers for DNA barcoding i.e. non-specific species amplification”.

It is important to note that although Linares *et al.* refer to LepF1/LepR1 as “Lepidoptera specific” primers, what was originally written was that LepF1/LepR1 was a “primer pair designed for Lepidoptera” [29]. In fact, it is clear from the intervening eight years since the initiating barcoding paper was published, through one million sequencing reactions at the Biodiversity Institute of Ontario using LepF1 or LepR1, that these primers have broad utility across most insect groups (from the publicly available BOLD website accessed on 11.04.19). Interestingly, Lineares *et al.* noted that, in spite of their concerns following discovery of *Wolbachia*, they did not find any “obvious association between host lineages and *Wolbachia* infections” (i.e. infection status did not appear to affect species identification via barcodes).

### Conflation of insect identifications due to the inclusion of the bacterial endosymbiont COI

To what degree is the non-specific amplification of *Wolbachia* COI a problem for the widespread application of DNA barcoding? It is apparent to us that it is exactly because barcoding is frequently successful for species identification that non-target amplification (between insect and bacteria) is not a major concern. It is immediately apparent when an endosymbiont COI fragment is unintentionally amplified from its host through the degree of difference between what was expected and what was generated (Table 3). It is because, vastly more often than not, barcoding can differentiate species that the inadvertent (and therefore mislabeled) inclusion of non-specific bacterial amplicons is not a major problem.

We do note that the majority of these extractions are made not from whole specimen or abdominal extractions but from legs. Although *Wolbachia* can be found in extractions made from somatic tissue, this is generally presumed to occur at a lower rate than for extractions made from the abdomen (however consider that the actual concentrations recovered by [21] were not different between reproductive and somatic tissue). Perhaps, our extraction protocol [30], produces, on average, more host DNA than the protocol followed by Linares *et al.* Alternatively, perhaps the high fidelity *Taq* (Platinum Taq DNA polymerase; Invitrogen) used in the Biodiversity Institute of Ontario permits the critical first stages of PCR to be swamped by the more abundant host DNA rather than that of the endosymbiont.

For example, consider the order Lepidoptera in general, and a specific case study of the ACG Lepidoptera [1] where we saw a very low rate of *Wolbachia* amplification. These low rates of non-intended amplification have not impeded the production of large numbers of Lepidoptera DNA barcodes, nor have we yet documented a case within the Lepidoptera where either the bacterial COI was confused for the insect, nor when differential possession of *Wolbachia* strain(s) has conflated population and species level divisions. Furthermore the non-intended amplification has produced some interesting ancillary findings. For instance, the two distinct *Wolbachia* COI sequences recovered from ACG Lepidoptera matched those of the MLST alleles coxA-1 and coxA-6, although since they do not completely overlap with the MLST standard coxA sequence, they may thus not be identical. The coxA-1 allele was primarily found in large butterflies and moths (Hesperiidae, Notodontidae and Nymphalidae) while the coxA-6 allele was found predominantly in smaller Pyralidae and Elachistidae. In addition, twenty-three (12%) of these bacterial contaminant sequences arose from the same host species (*Caligo telamonius* Felder, Nymphalidae).

### Conflation of infected lineages with species via the effects of *Wolbachia*

Due to the heightened capacity for these bacteria to fragment the mitochondrial lineages of a species, concern has been expressed regarding what impact their apparent omnipresence has on a mitochondrial system of DNA-based species identification and discovery [31]. Specifically, problems will arise if more lineages than are truly present are flagged as new or different species as a result of *Wolbachia* separated mtDNA lineages harbored within a single species (a statistical Type I error (rejecting a true null when the initial null hypothesis is that specimens are of the same species)).

Alternatively, *Wolbachia* infections can sweep away the mitochondrial variation between species – if even infrequent hybridization events result in the fixation of the endosymbiont. In one recent example, the lack of within-species monophyly was hypothesized to result from introgressive hybridization associated with *Wolbachia* infection [12]. Similar patterns of evident interspecific mitochondrial introgression have been noted in sister species of parasitic wasps [20], butterflies [32] and *Drosophila* [33]. However, it is not clear from the literature how common this is (e.g. “We see no obvious association between host lineages and *Wolbachia* infections” [13]). From the perspective of our dataset, we have seen no evidence of this type of between-species mtDNA barcode sharing due to the sharing of *Wolbachia* infections – with one possible exception.

The one example where there was an apparent mito-nuclear discordance – possibly caused by *Wolbachia* – was documented in the Costa Rican tachinid fly, *Chetogena* scutellarisDHJ01 [11]. The presumably generalist (polyphagous) tachinid “*Chetogena scutellaris*” was found to include two barcode groups: *C.* scutellarisDHJ01 and *C.* scutellarisDHJ02. Both groups were also supported by divergences within 28S and ITS1. However, within *C.* scutellarisDHJ01, there was an additional rDNA split that was not apparent in the barcode. Using the *wsp* assay it was found that nearly ¾ of the specimens of *C.* scutellarisDHJ01 contained *Wolbachia* – and thus suggested that *Wolbachia* may have been the source that swept mtDNA variation from this provisional morphologically cryptic species that is nonetheless diagnosable with nuclear sequences.

*Wolbachia* infections can also inflate the estimates of intra-specific diversity – if different strains infect different populations or individuals within a population. One example where there were evidently different *Wolbachia* strains present in different provisional and morphologically cryptic species was described recently [7]. Here, one apparent morphospecies of *Pristomyrmex* was collected from a threatened population. Specimens from these collections were found to contain deep barcode divergences (15%) suggesting the morphospecies actually contained multiple cryptic species, or that the population may be a contemporary refuge for two apparently divergent mtDNA lineages. One of two rDNA loci tested revealed corroborating variation and all *Pristomyrmex* specimens tested positive for *Wolbachia*. However, each provisional species was infected with different *Wolbachia* strains – suggesting that the presence of the different strains of endosymbiont alone could have produced the evident patterns of mitochondrial divergence. It is clear that these provisional *Pristomyrmex* species harbor different *Wolbachia*, and it is also possible that the infection with different strains of *Wolbachia* has played a role in the evident diversification within these cryptic species. Shoemaker *et al.* [34] and Sun *et al.* [35] also discuss speciation events within host insect species of *Drosophila* and *Eupristina* that were putatively reinforced by a *Wolbachia* infection. In this case, we observed that the *wsp* sequences from one provisional *Pristomyrmex* species contained multiple peaks, while the *wsp* from other provisional species had unambiguous base pair callings. This suggests that rather than unidirectional cytoplasmic incompatibility (CI: prevention of intra-lineage mating through presence/absence of a *Wolbachia* strain), this *Pristomyrmex* example may be driven by the prevention of intra-lineage mating through the possession of different strains (bidirectional CI).

In another example, we examined intraspecific divergences in ant species of Mauritius [7] that were infected or uninfected with *Wolbachia*. The published supporting information file for this dataset contains information regarding the infection status per individual and species based on the *wsp* assay (http://www.frontiersinzoology.com/content/6/1/31/additional). Using this coding, we searched for infected or uninfected species from the public BOLD project “Ant Diversity of Mauritius (ASMA)” (when individuals from a species had been recorded as both uninfected and infected individuals, the species was coded as infected in this analysis). For each infection status, we then used BOLD to calculate distance summary statistics (Table S1). While the *Wolbachia* infected species contained slightly less variation, the difference was slight (the average intra-specific distance for *Wolbachia* infected species is 0.824, while for uninfected it is 0.99). While these results need be understood as preliminary and ought not to be generalised as they arise from one taxonomic case on an isolated island, they are nevertheless demonstrative of the capacity to identify insect species in spite of *Wolbachia* infection and furthermore, the capacity to use somatic DNA extractions to study a species' *Wolbachia* infections (specifically when there are multiple specimens sequenced per species).

### Amplification and Primer Design

**Figure 2:**
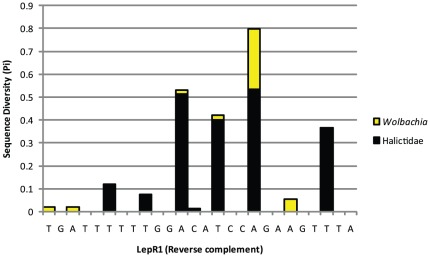
The nucleotide diversity of region of the LepR1 oligonucleotide as compared to GenBank Halictidae COI sequences (156 sequences from 93 species in black) and MLST *Wolbachia* database sequences (104 sequences in yellow) was calculated using DNASP [**45**]. It is clear that the reverse primer (LepR1) is better fit to the bacterial endosymbiont than to the insect host. Specimen information for each data set is included in Table S2.

It is clear that *Wolbachia* COI can be amplified from DNA extractions of insects made from somatic tissue [21]. However, in our data, the frequency of this occurrence within the case study projects (min of 0%, max 0.61%, mean 0.12% - Table 3) suggests that this does not compromise the barcoding of their arthropod hosts, nor de facto require the design of genus-specific COI primers [13]. While such re-design is not required in general, it may be necessary in some cases. The difference in the proportion of amplification between different groups of insects is of interest. For instance the halictid bees, including the largest (>1,750 spp) and perhaps most taxonomically challenging bee genus (*Lasioglossum*), appear to contain a relatively high preponderance of *Wolbachia*. This may be due to an increased infection load (and therefore increased likelihood of infection due to that ‘super-infection’ load being carried by the individual insect) or, alternatively, lack of fit for the near-universal insect COI primers within this specific family (if the target insect COI is not amplified in the important initial stages of PCR, the proportion of co-amplifying endosymbionts becomes more important). In a subset of the bee data (570 specimens), ten *Wolbachia* COI sequences were produced using LepF1/LepR1. However, re-amplification from the same extracts using the same primers but paired with degenerate internal primers (C_ANTMR1D and RonMWASPdeg_t1 respectively) produced the bee mtDNA barcode in all cases. While the use of a degenerate primer cocktail does not preclude the amplification of bacterial COI (Table 2), it did reduce the frequency of bacterial amplification for these bees. When the fit of one of the standard near-universal insect primers (LepR1) was compared to Halictidae in GenBank, and the *Wolbachia* MLST strain database, it is apparent that the LepR1 primer has a much better fit with the bacterial endosymbiont than with the insect host (Figure 2). Halictidae represents a case where family specific primer design is warranted.

### *wsp* assay COI amplification and *Wolbachia* prevalence

Standard protocols for *Wolbachia* screening usually call for fresh abdominal tissue from the insect host, while insect DNA barcoding is more typically done by sampling a leg from a preserved specimen. Due to this difference alone, routine *Wolbachia* screening on barcoded specimens will likely miss some true infections, and therefore underestimate infection rates [7]. However, our results suggest that integrating the two sampling surveys would likely provide access to an abundance of previously un-anticipated diversity.

In all cases (ants from Mauritius and ants and parasitoid wasps of Churchill, Manitoba, Canada) our examples support the hypothesis that many more of these insect specimens and species carry *Wolbachia* than are apparent by our inadvertent COI bacterial amplification, a finding in agreement with other studies [36].

In addition to comparing recovered bacterial COI to wsp surveys, for one group we used the literature to calibrate our finding of inadvertent endosymbiotic COI amplification. For fig wasps, the prevalence of *Wolbachia* COI revealed in the barcoding assay was large compared to the other test datasets analysed here (∼9%). Yet when calibrated to the overall expected prevalence of *Wolbachia* known from Chinese fig wasps (∼50% in all species [37], [38]) this value appears low. Within one hesperiid genus of ACG Lepidoptera (Urbanus) we amplified a wsp gene fragment that was identified as Supergroup B. Within this genus we have never inadvertently amplified bacterial COI and this case is the only incidence where *Wolbachia* strains from Supergroup B have yet appeared in our data (although it should be noted that, due to recombination, the use of wsp alone to categorise Supergroup ought to be interpreted with caution [39]).

### COI allele group diversity

We compared the fragments of isolated *Wolbachia* COI that we generated to the MLST database for *Wolbachia* that includes COI as one of the six loci used for typing the strains of this bacterium (coxA in MLST terminology). However, it is important to note that the accepted MLST COI fragment is in the 3′ region of the gene and has very little overlap (194 bp) with the standard barcode locus. Despite this small degree of overlap, there was sufficient variation to compare the COI alleles from the MLST to the COI fragments fortuitously generated here. We found that the majority of the diversity fell within Supergroup A and, while some strains appear novel, most were associated with existing strain types; however a thorough comparison of databases would require congruent COI regions.

### Geography and Genetic Isolation by Distance

**Figure 3:**
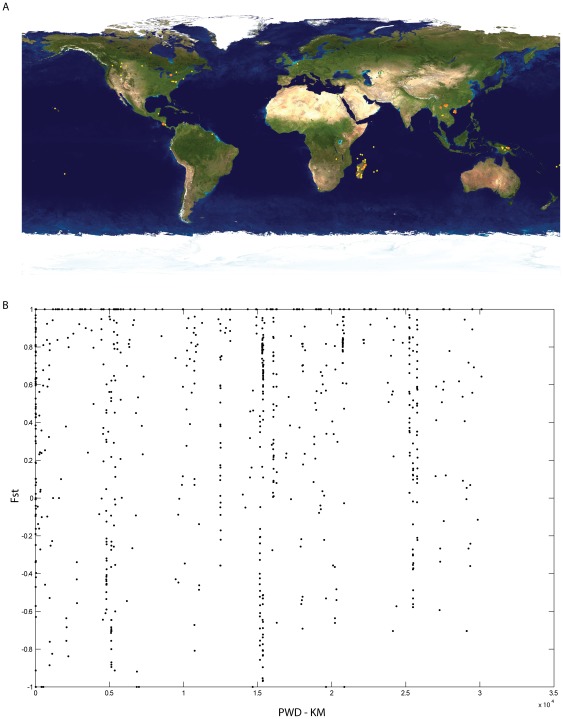
Insect host geographic distribution A) Red and yellow dots indicate the collection locality for *Wolbachia* insect hosts. **B**) Mantel test of pairwise Fst of *Wolbachia* COI and kilometers for the collection localities of insect hosts (r = 0.099, p = 0.92).

In ants, *Wolbachia* strains from New World collections were shown to differ from those in ants from elsewhere [40]. When compared across all host families we detected no evident pattern of isolation by distance in the bacterial COI gene (Figure 3). Within COI, we found slight COI divergences (∼1%) between the *Wolbachia* from ants across Old and New World. For instance, *Wolbachia* COI from a Costa Rican ant differed by 2 and 4 base pairs respectively to those from bacteria hosted by ants in Papua New Guinea and Mauritius. While the COI region alone does not appear to have sufficient resolution to observe the patterns of New World/Old World divergence described in [40], within the more variable wsp, we did see, on average, 17% divergence between ants from Mauritius and Churchill, Manitoba Canada. As a comparison, the *Wolbachia* wsp of Costa Rican tachinid flies and Mauritus ants was found to be only 9% divergent. Patterns of evident isolation by distance in *Wolbachia* must be approached with caution – and calibrated with information from more than one insect host family.

### Conclusions

Our results suggest that insect barcoding is not compromised by the presence of *Wolbachia*. Insect DNA barcodes are easy to differentiate from the sequences of their bacterial endosymbionts in cases when inadvertent amplification occurs and, based on several hundred thousand amplifications, the bacterial sequences do not occur frequently. However, insect barcoding projects would do well to incorporate additional steps that standardize the collection of the ancillary data present in whole genome extracts, including *Wolbachia* MLST analyses – and in increasing the number of extractions based on abdomens rather than somatic tissue. This would help both to document our expectations regarding the prevalence of this bacterium and to explain unanticipated patterns of mitochondrial sharing or divergence. In addition – the *Wolbachia* MLST program would also benefit from expanding and/or shifting the COI region included in its database to overlap with the large (at writing >1.25 million records) database of eukaryotic COI sequences. Expanding the current MLST standardized selection of COI to align with the eukaryotic DNA barcoding region would permit a more thorough comparison of mitochondrial diversity, even though it is evident that the great majority of *Wolbachia* infections will go unnoticed in standard COI barcoding protocols. Such standardization would help explain the apparently new allele groups recovered here (particularly when the insect portion of BOLD could be positioned to be a major contributor to the MLST campaign). The *Wolbachia* COI alleles seen here are broadly distributed geographically and, with some exceptions within the ants, strain type does not appear to be tightly associated with their hosts. While preliminary, our results demonstrate the benefits and potentials of integrating *Wolbachia* surveys into insect DNA barcoding projects. In understanding the species within ecological communities, we would do well to understand the communities within those species [41].

## Methods

After being given special access to all traces files produced by the Canadian Center for DNA Barcoding on the BOLD database, we scanned nearly 2.2 million trace files for matches to *Wolbachia* COI sequences by blasting trace sequences to a *Wolbachia* COI reference library. The reference library was constructed from single representatives of each strain in GenBank where COI sequences of sufficient length were available. Traces were matched to the reference library based on an e-value threshold of <1e-110 (Figure S1).

A query for *Wolbachia* COI traces was possible for this survey because BOLD preserves all electropherograms produced for every individual record even if the sequence itself is identified as a contaminant and excluded from the database as a result of the quality-control procedures in place, which includes screening for sequencing of non-target COI *Wolbachia* amplicons.

All COI fragments were generated using standard extraction and amplification protocols at the Biodiversity Institute of Ontario [30], [42], [43]. Primers utilized for generating COI are standard barcoding primers that are listed in Table 2.

*Wolbachia* COI fragments were each assigned a sample ID number that corresponded to the BOLD process ID number of the host DNA extract with a suffix of “.w” attached. Thus, the *Nesomyrmex* ant sample CASENT0152435-D01 can be accessed through Antweb by this accession, or BOLD as ASANV619-09, while the bacteria associated with the ant specimen can be accessed by ASANV619-09.w. All *Wolbachia* COI sequences generated here are available on BOLD within the container project: Insect Endosymbionts (ASENZ) and on GenBank. All accession numbers and insect collection details are available in Table S2.

For four subsets of the data, we used the PCR based *wsp* assay [22] to determine the proportion of insect specimens that were infected. We compared this rate of *wsp* determined prevalence to the rate at which bacterial COI had been produced from insect leg extractions (Table 3). For a sub-set of these positives, we amplified the *wsp* product to confirm its identity.

The Mantel test, measuring isolation by distance on bacterial COI was completed using Arlequin v3 [44] where geographic distances was based on the insect host collection locality.

Measures of diversity at each site within the oligonucleotide for LepR1 were completed using DNASP [45].

## Supporting Information

Figure S1 **The number of trace files matching** ***Wolbachia*** **in BOLD trace library.** The value of <1e-110 was chosen as a threshold between the conservative match of query to known *Wolbachia* strains and the identification of novel strains.
(PDF)

Table S1 **DNA barcode diversity for ant species of Mauritius coded by** ***Wolbachia*** **infection.**
(XLS)

Table S2 **Collection details, accessions (BOLD and GenBank) and all host information associated with** ***Wolbachia*** **DNA sequences used here.**
(XLS)
