# Supplementary figures and images for: Wolbachia and DNA Barcoding Insects: Patterns, Potential, and Problems
Source: PLoS One. 2012 May 2;7(5):e36514. doi: 10.1371/journal.pone.0036514 (PMC3342236; doi:10.1371/journal.pone.0036514)

**Figure S1.**

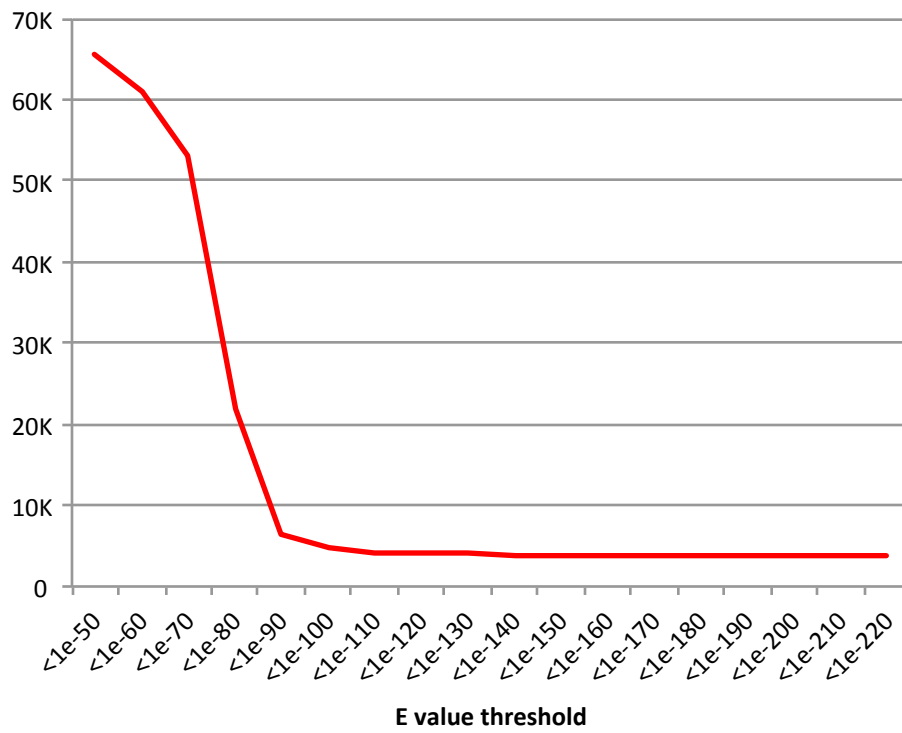

Supplement: Figure S1 — The number of trace files matching Wolbachia in BOLD trace library. The value of <1e-110 was chosen as a threshold between the conservative match of query to known Wolbachia strains and the identification of novel strains. (PDF) [file pone.0036514.s001.pdf]
